# Morphology and Composition of Lumbar Intervertebral Discs: Comparative Analyses of Manual Measurement and Computer-Assisted Algorithms

**DOI:** 10.3390/bioengineering11050466

**Published:** 2024-05-08

**Authors:** Yiting Cheng, Yuyan Ma, Kang Li, Celal Gungor, Richard Sesek, Ruoliang Tang

**Affiliations:** 1 School of Mechanical Engineering, Sichuan University, Chengdu 610000, China; chengyit1@163.com; 2 Sichuan University-Pittsburgh Institute (SCUPI), Sichuan University, Chengdu 610000, China; myuyan2022@163.com; 3 West China Biomedical Big Data Center, West China Hospital, Sichuan University, Chengdu 610000, China; likang@wchscu.cn; 4 Department of Forest Industrial Engineering, Izmir Katip Celebi University, Cigli 35620, Turkey; celal.gungor@ikc.edu.tr; 5 Department of Industrial and Systems Engineering, Auburn University, Auburn, AL 36849, USA; sesek@auburn.edu; 6 Nursing Key Laboratory of Sichuan Province, Chengdu 610000, China

**Keywords:** computer-assisted segmentation, fuzzy C-means, region growing, lumbar spine, intervertebral disc, nucleus pulposus, cross-sectional area, finite element modeling

## Abstract

Background: The morphology and internal composition, particularly the nucleus-to-cross sectional area (NP-to-CSA) ratio of the lumbar intervertebral discs (IVDs), is important information for finite element models (FEMs) of spinal loadings and biomechanical behaviors, and, yet, this has not been well investigated and reported. Methods: Anonymized MRI scans were retrieved from a previously established database, including a total of 400 lumbar IVDs from 123 subjects (58 F and 65 M). Measurements were conducted manually by a spine surgeon and using two computer-assisted segmentation algorithms, i.e., fuzzy C-means (FCM) and region growing (RG). The respective results were compared. The influence of gender and spinal level was also investigated. Results: Ratios derived from manual measurements and the two computer-assisted algorithms (FCM and RG) were 46%, 39%, and 38%, respectively. Ratios derived manually were significantly larger. Conclusions: Computer-assisted methods provide reliable outcomes that are traditionally difficult for the manual measurement of internal composition. FEMs should consider the variability of NP-to-CSA ratios when studying the biomechanical behavior of the spine.

## 1. Introduction

Human spinal intervertebral discs (IVDs) are located between two adjacent vertebral bodies and enclosed by ligaments anteriorly and posteriorly. Together, they form the major joints of the spine, providing its structural integrity and mobility. In general, healthy spinal IVDs are complex structures comprised of three distinct components, the outer fibrocartilaginous annulus fibrosus (AP), the central proteoglycan-rich nucleus pulposus (NP), and two cartilaginous vertebral endplates (EP) superiorly and inferiorly. Each component has distinctive structural compositions, contributing to unique properties and responses to mechanical loadings. Therefore, modern comprehensive approaches, such as finite element modeling (FEM), that are used to characterize the mechanical behaviors of the human spine rely on accurate geometric and property data of all finite elements to better investigate their mechanical behaviors and study the associated failure modes and underlying mechanisms leading to spinal pathologies [1]. On the other hand, the vast diversity and high variability in spinal geometry among the general population should also be considered, especially when developing both generic and individual-specific FEM models [1,2,3,4,5]. In general, vertebral body size, IVD height, and cross-sectional area (CSA) are commonly reported geometric parameters with significant influence on FEM model performance and outcomes, such as axial displacement, pressure distribution, morphological changes, and the risk of disc bulge [2,3,4,5]. These external morphological dimensions are peripheral and relatively easy to approach and measure [6,7]. On the contrary, the internal structures and compositions of spinal IVDs have not yet been comprehensively measured and fully investigated, particularly the NP-to-CSA ratio and its contribution to the mechanical properties of spinal motion segments compared to peripheral/external dimensions (e.g., width, depth, CSA, etc.) [7]. A recent summary of previous FEM studies on spinal IVDs and motion segments revealed a relatively large discrepancy (i.e., 21% to 60%) in the NP-to-CSA ratios either reported or referred to in the literature [6]. This is in line with previous understandings of spinal morphology that the lumbar nucleus, in general, takes about 30% to 50% of the total disc area [8]. The scarce geometric data on the internal structures identified in the current body of literature have been primarily derived from manual measurements performed during autopsy [8]; although, most of these studies, unfortunately, did not provide clear and detailed descriptions of the measurement protocol or report the assessment of measurement reliability, resulting in substantially limited reference value for subsequent investigations [7]. Moreover, these autopsy-derived measurement results, while being highly susceptible to post-mortem changes and complications during the specimen preparation [9,10], may not be representative of the general and healthy population [7]. In addition, there is also a lack of understanding regarding the potential associations between the internal structural dimensions and other factors, such as spinal levels, gender, and anthropometric characteristics, etc., as such correlations have been reported in previous morphological studies of the peripheral/outer IVD dimensions [7,11,12].

Magnetic resonance imaging (MRI) technology, as a non-ionizing modality, is a rapidly developing clinical diagnostic method and provides the most comprehensive non-invasive evaluation of a greater number of spinal abnormalities owing to its multi-planar capabilities and superior soft tissue contrast [13]. In addition, MRI scans are also an ideal candidate for image-based applications involving IVD anatomy, geometry, and composition [7,14]. Clustering-based segmentation is a type of unsupervised segmentation method with high efficiency and better accommodation for untrained low data. Common clustering algorithms, such as fuzzy C-means (FCM) and region growing (RG), have been widely used in a variety of soft tissue segmentation applications (e.g., blood vessels, organs, tumors) [15,16,17], while the RG algorithm has been proven effective in medical image segmentation, including CT and MRI scans, where a clear boundary can be identified between two regions [18,19,20]. Computer-assisted image segmentation has also been used in morphological applications involving musculoskeletal tissues, such as the spine and paraspinal muscles [7,11,21,22,23]. However, previous investigations of spinal morphology have mainly focused on the peripheral/external dimensions.

Therefore, this study focused on the morphological characteristics of distinctive IVD structures and components using MRI scans. The purpose of the present study was to (1) establish an objective measurement protocol for the intrinsic structures and compositions of spinal IVDs in the transverse section and (2) investigate the potential influence of individual factors, such as gender and spinal level.

## 2. Materials and Methods

The MRI data used in this study were derived from two previous studies based on (1) archived medical records (AMRs) [7] and (2) scans of asymptomatic subjects (ASYs) [11], respectively. All MRI data were screened, anonymized, and transported in digital imaging and communications in medicine (DICOM) format. Research methods and protocols used in these two studies were approved by their corresponding IRBs and are described in detail elsewhere [7,11]. A brief description of these methods is provided below. This study was approved by the Institutional Review Board (IRB) at Sichuan University.

### 2.1. Populations

#### 2.1.1. Archived Medical Records (AMRs)

MRI scans were obtained from the AMR database at the University of Utah Hospital (Salt Lake City, UT, USA) and included a total of 87 subjects (44 F and 43 M) between the ages of 20 and 40 years who had undergone spinal MRI scans performed on a 1.5 T scanner (Siemens MAGNETOM Avanto, Siemens AG, Erlangen, Germany) in a headfirst supine position. All MRI data (i.e., T2-weighted) were collected with parameters that supported morphometric analyses (i.e., repetition time ranged from 3000 to 4770 ms, echo time between 80 and 110 ms, and section thickness between 3 and 4.5 mm). Subject demographic data (e.g., height, weight, age, and gender) were embedded and released along with the DICOM data. Initial MRI scans were reviewed based on the corresponding radiology reports to exclude subjects with (1) positive diagnosis of specific low back disorders, (2) evidence of morphological alterations in the lumbar or thoracic spine (e.g., collapsed disc, trauma), and (3) any known pathology relevant to and likely to alter the spinal geometry (e.g., scoliosis, tumors).

#### 2.1.2. Asymptomatic Subjects (ASYs)

A cohort of 35 subjects with no current symptoms of back disorders (13 F and 22 M) was recruited from the study body at Auburn University and scanned by a 70 cm Open Bore 3 T scanner (MAGNETOM Verio, Siemens AG, Erlangen, Germany) at the Auburn University MRI Research Center using a standard morphological T2-weighted turbo-spin-echo (TSE) sequence with a repetition time of 4400 ms, echo time of 100 ms, and matrix of 320 × 240. The section thickness was 3 mm and the voxel size was 0.69 mm × 0.69 mm × 3 mm. Each subject, in a headfirst supine position, was scanned twice, following two sequence protocols. The first protocol scanned the lumbar region with standard horizontal slices and the second protocol scanned the same lumbar region using oblique slices parallel to each IVD and the two associated endplates to minimize distortion. Therefore, ASY subjects contributed two sets of MRI images. No subjects reported any back problems for the previous two years and had never received any medical treatment for back pain.

### 2.2. Measurement of IVD Structures

All IVDs were then assessed for health status by a clinical radiologist using the Pfirrmann grading system [24]. IVDs with Grade IV and V status were excluded from the subsequent measurement due to the lack of distinction between NP and AF structures caused by disc degeneration and collapsed intradiscal space [24]. However, it should be noted that exclusions were only applied to specific IVDs, not to the corresponding subjects. In other words, if one subject had Grade V L5/S1 IVDs but Grade I L3/L4 and L4/L5 IVDs, only the L5/S1 IVDs were excluded. Therefore, the number of subjects studied at each lumbar level varied. In total, 123 subjects (58 F and 65 M) were included, resulting in a total of 400 IVDs. 

All MRI scans were analyzed using open-source DICOM software Osirix (version 12 Lite; 32-bit) [25]. All image analyses were performed using MATLAB (Version 2021a, MathWorks, Natick, MA, USA).

#### 2.2.1. Tracing the IVD Contours

**Figure 1:**
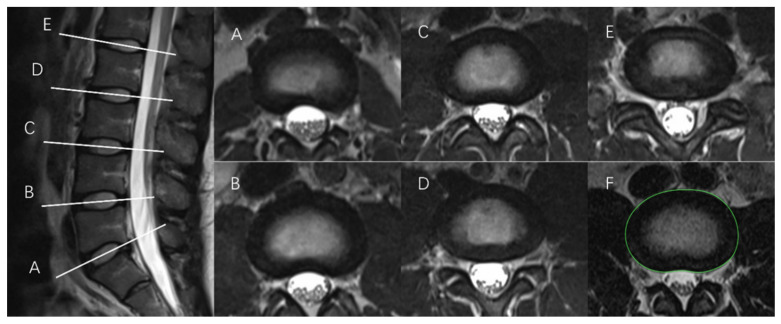
T2-weighted MRI scans of the sagittal lumbar spine at L5/S1 (**A**), L4/L5 (**B**), L3/L4 (**C**), L2/L3 (**D**), and L1/L2 (**E**) (left) and corresponding images (i.e., the same alphabet letter) in the transverse plane for the measurement of cross-sectional area (right) (note: subfigure (**F**) illustrates the outcome of IVD contour tracing, highlighted with the green line).

First, the outer peripheral contour of each IVD was determined and expressed as the cross-sectional area of the IVD (i.e., CSA_IVD_). In this study, CSA_IVD_ data were retrieved from previous investigations, where detailed measurement protocols are described elsewhere [7,11]. In general, at each level, the IVD contour (i.e., outer perimeter) was manually identified and traced in the transverse section, using an optical mouse at a computer workstation with a high-definition monitor (i.e., display resolution 2560 × 1440). The specific transverse MRI scan was identified when, in the corresponding sagittal section, it was evident that the scan was taken through the center of the IVD, as shown in Figure 1 (left). Then, the corresponding MRI scan with the contour trace was saved in JPEG format and kept on file for subsequent measurements. The contour was marked with a significant color, as shown in Figure 1 (right). Marks were taken only in oblique slices to minimize distortions.

#### 2.2.2. Tracing the NP Contours

**Figure 2:**
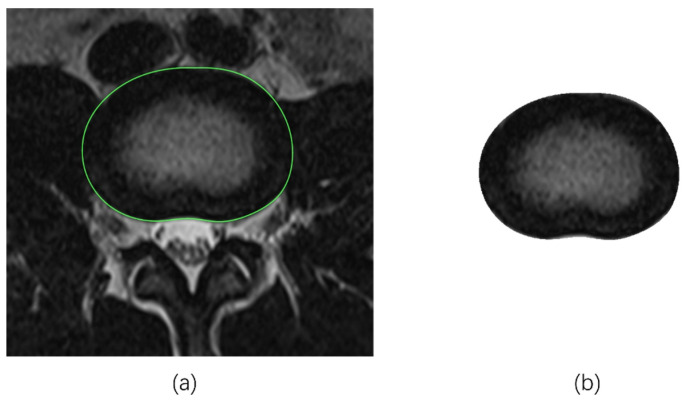
Example of the preparation of IVD image for segmentation process using K-means method ((**a**). green line highlights the IVD contour with respect to the surrounding structures; (**b**). IVD image cropped to only maintain the AP and NP components).

First, each saved image with an IVD contour in the transverse section was cropped to exclude the surrounding structures and tissues to retain the IVD itself (i.e., only the AP and NP components) using the K-means clustering algorithm (K-means) (Figure 2). Then, each processed IVD image was prepared for two segmentation algorithms, fuzzy C-means (FCM) and region growing (RG), respectively, as follows.

- Fuzzy C-means algorithm (FCM)

FCM is a division-based clustering method that is an improvement upon the traditional C-mean algorithm. The idea of FCM is to make objects classified in the same cluster have maximum similarity and objects in different clusters have the least similarity [26]. Compared with the hard division of the traditional C-mean algorithm, FCM uses a flexible fuzzy division. Therefore, it has advantages in medical image processing with noise and can obtain more accurate segmented images. 

FCM partitions set n objects $x=\left\{x_{1},x_{2},x_{3}\ldots x_{n}\right\}$ in $R^{d}$ dimensional space into c(1<c<n) fuzzy clusters with cluster centers or centroids. The fuzzy clustering of objects was described by fuzzy matrix U with n rows and c columns, where n is the number of data objects and c is the number of clusters. $u_{ij}$ is the element in the $i^{th}$ row and $j^{th}$ column of the matrix, which represents the association degree between the $i^{th}$ vector object and the $j^{th}$ cluster, and the association degree takes a value between 0 and 1. Briefly, the process of FCM is the process of minimizing the objective Function (1):

(1) $$J\left(U,c_{1},\ldots ,c_{c}\right)=\sum_{i=1}^{c}J_{i}=\sum_{i=1}^{c}\sum_{j}^{n}U_{ij}^{m}d_{ij}^{2}$$

where $d_{ij}=||c_{i}-x_{j}||$ is the Euclidean distance between the $i^{th}$ cluster center and the $j^{th}$ data point; the fuzziness of the obtained clusters is controlled by m, called the weighted index, which is a scalar quantity; $c_{i}$ refers to the center of mass of the $i^{th}$ cluster, which is represented by Function (2):

(2) $$c_{i}=\frac{\sum_{j=1}^{n}u_{ij}^{m}x_{j}}{\sum_{j=1}^{n}u_{ij}^{m}}$$

**Figure 3:**
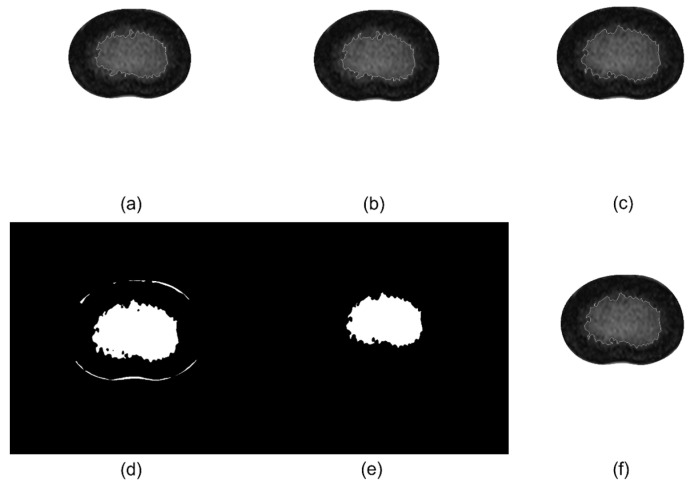
Examples of the FCM process: (**a**) the 1st cluster obtained; (**b**) the 6th cluster obtained after 5 iterations; (**c**) the cluster obtained after 20 iterations; (**d**) the final cluster obtained, shown as mask, containing interference clusters; (**e**) the removal of interference clusters using open operations; and (**f**) the final clustering results.

FCM clustering algorithm is an iterative process (Figure 3). The implements of FCM are as follows:

(a) Initialize the affiliation matrix U with random numbers between 0 and 1 so that it satisfies the constraint in Equation (3):
  (3) $$\sum_{i=1}^{c}u_{ij}=1,\forall_{j}=1,\ldots ,n$$
(b) Obtain the $c^{th}$ cluster centers $c_{i},(i=1,2,3,\ldots ,c$) using Equation (2).
(c) Calculate the Euclidean distance $d_{ij}=||c_{i}-x_{j}||$ between the $i^{th}$ cluster center and the $j^{th}$ data point.
(d) The algorithm stops if the value function obtained by Equation (1) is less than a determined threshold value or if the change of the value function relative to the last iteration is less than the threshold value. The algorithm skips to step f.
(e) Use Equation (4) to compute the new U matrix; then, return to step b and keep iterating.
  (4) $$u_{ij}=\frac{1}{\sum_{k=1}^{c}{\left(\frac{d_{ij}}{d_{jj}}\right)}^{2/(m-1)}}$$
(f) Output clustering center $c_{i}$ and the affiliation matrix U [1].

- Region growing algorithm (RG)

**Figure 4:**
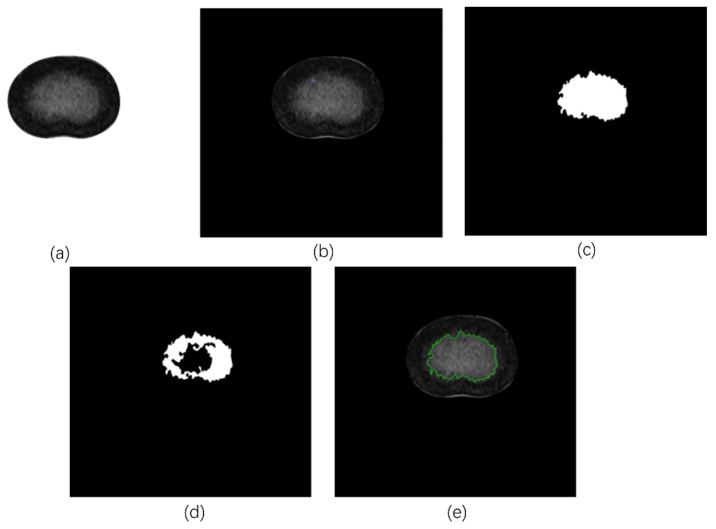
Example of RG process: (**a**) initial image identified for processing; (**b**) selection of the initial seed points; (**c**) resulting mask for maxdis = 0.1; (**d**) resulting mask for maxdis = 0.06; and (**e**) final result (green line highlights the NP contour).

The basic idea of region growing is to gather pixels with similar properties to form a region. Specifically, a seed pixel is found as the starting point and then the seed pixel and the pixels in the surrounding neighborhood that have the same or similar properties to the seed pixel are merged into the region where the seed pixel is located until no pixel satisfies the criterion (Figure 4). The region growing is terminated, thus achieving the extraction of the target [18,27].

The steps to achieve region growing are as follows:

(a) Select the initial pixels based on the nature of the image. In the case of this nucleus pulposus segmentation, because the boundary of the NP is blurred and the gray value near the boundary is significantly lower than that at the center of the NP, the point close to the boundary of the NP is selected as the seed pixel. This choice has shown better results in practice. The center of the NP is not the seed pixel because sometimes the NP contour cannot be obtained from the center of the NP.
(b) Set $\left(x_{0},y_{0}\right)$ as the center and add its four neighboring pixels into the stack to be scanned (known as Seeds), whose coordinates are$\;(x_{0}-1,y_{0}),(x_{0}+1,y_{0}),(x_{0},y_{0}-1)$, and $\left(x_{0},y_{0}+1\right)$. The growth criterion is interpreted as pixel t(x,y) from Seeds to make the difference between the gray value of this point and the mean gray value of the segmented area the smallest.

If (x,y) does not exceed the image boundary and satisfies the growth criterion, divide (x,y) and $\left(x_{0},y_{0}\right)$ into the same region and add the 4 neighboring pixels of (x,y) to Seeds. Then, calculate the new mean gray value of the region:
  (5) $$mean2=\frac{mean1\times size+point}{size+1}$$
  where mean2 denotes the new mean, mean1 denotes the old mean value, size indicates the number of pixel points in the segmented area, and point indicates the gray value of the current newly selected point.

(c) Take a pixel from Seeds and treat it as the initial pixel $\left(x_{0},y_{0}\right)$ and return to step 2 for iteration.
(d) When Seeds is empty, return to step 1.
(e) Repeat steps 1 through 4, when the gray value distances of all the neighboring pixels to be analyzed and already segmented in Seeds are all greater than the pre-set threshold (i.e., maxdis), the region growing ends.
(f) Perform expansion corrosion and opening–closing operations on the obtained region, draw the outline of the obtained mask, and complete the extraction of the NP region.

**Figure 5:**
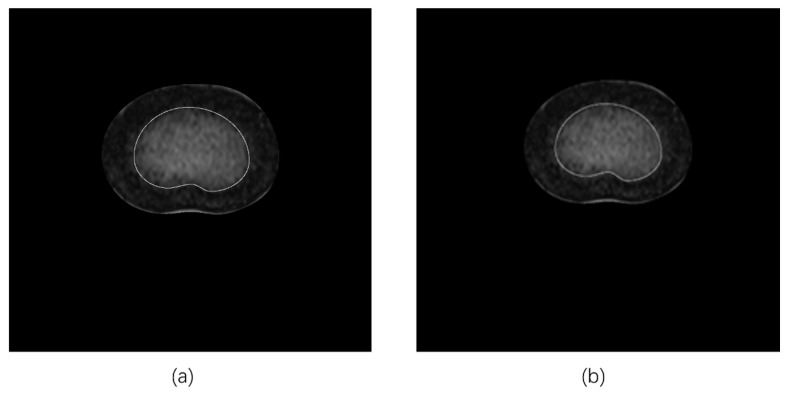
Morphological constraints on the results using the specific parameter equation: (**a**) FCM; (**b**) RG.

The NP outer profiles obtained through FCM and RG algorithms, respectively, were further optimized (Figure 5), using a set of parametric equations to improve the algorithm and keep the basic morphological structures, (i.e., the transition from oval-shaped in the upper lumbar to kidney-shaped in the lower lumbar).

- Manual tracing (MT)

To evaluate and compare the results of computer-assisted algorithms, a subset of MRI images (n = 21 out of 105; 20% of the MRI dataset) was randomly selected to be manually measured by a spine surgeon with 10 years of clinical practice. This surgeon was asked to manually trace and label the NP contours. After one week, the same surgeon performed another round of measurements. The results were used to calculate the manually determined NP-to-CSA ratios and assess the repeatability of the measurement.

### 2.3. Determination of NP-to-CSA Ratios

In this study, the NP-to-CSA ratios were calculated and expressed as a percentage obtained by dividing CSA_NP_ by CSA_IVD_ (Equation (6)):

(6) $${Ratio}_{NP-to-CSA}=\frac{{CSA}_{NP}}{{CSA}_{IVD}}\times 100$$

### 2.4. Data Analysis

Split plot factorial (SPF) analysis of variance (ANOVA) was used to analyze the effect of gender (i.e., 2 levels) and spinal level (i.e., 4 levels; L1/L2, L2/L3, L3/L4, and L4/L5) on the results. Paired sample *t* test and Pearson’s correlation coefficient (PCC) were used to determine the repeatability of manual measurement and interpreted according to previous studies (i.e., Excellent: PCC > 0.810; Good: [0.61, 0.809]; Moderate: [0.410, 0.609]; Fair: [0.210, 0.409]; and Poor: PCC < 0.209) [28,29]. Absolute error was determined and expressed as the absolute difference between the two manual measurements divided by the first one. Note that manual results were based on the average of two manual measurements and used in subsequent analyses. Paired sample *t* tests, intra-class correlation coefficients (ICCs), and Pearson’s correlation coefficients (PCCs) were used to compare data between the two methods. Tukey’s honest significant difference (HSD) post hoc tests were used to determine the trend of change in ${Ratio}_{NP-to-CSA}$ with respect to spinal levels. All data analyses were conducted using R statistical software for Windows (version 4.3.2, The R Foundation for Statistical Computing, Vienna, Austria). An alpha level of 0.05 was established for all statistical tests.

## 3. Results

Table 1 provides a summary of the demographic data for subjects included at each lumbar level. After applying the exclusion criteria, the actual number of MRI images analyzed at each level was different. Therefore, it was determined that the corresponding subject demographic data should be presented. One more male subject was also included at L1/L2, L2/L3, and L3/L4, despite the missing demographic data. In this study, male subjects were significantly taller and heavier than females (*p* < 0.05).

### 3.1. Repeatability of Measurement

With respect to manual measurement, the intra-observer reliability was found to be good (PCC = 0.645). However, significant differences were found between the NP-to-CSA ratios derived from the first and second measurements (mean = 4%; *p* < 0.001). The average absolute error between the two results was found to be 10.5% (i.e., 10.5 ± 12.51), with a minimum of 0.01% and a maximum of 56.5%.

### 3.2. Comparison of Results Derived from Different Methods

Based on the randomly selected subset of MRI images, correlation analyses revealed excellent agreement between the manual and FCM methods (PCC = 0.845, *p* < 0.001) and good agreement between the manual and RG methods (PCC = 0.549, *p* = 0.010) and between the FCM and RG methods (PCC = 0.778, *p* < 0.001). Results from ANOVA (Table 2) revealed a significant influence of the method used to determine the NP-to-CSA ratio (*p* < 0.001).

**Figure 6:**
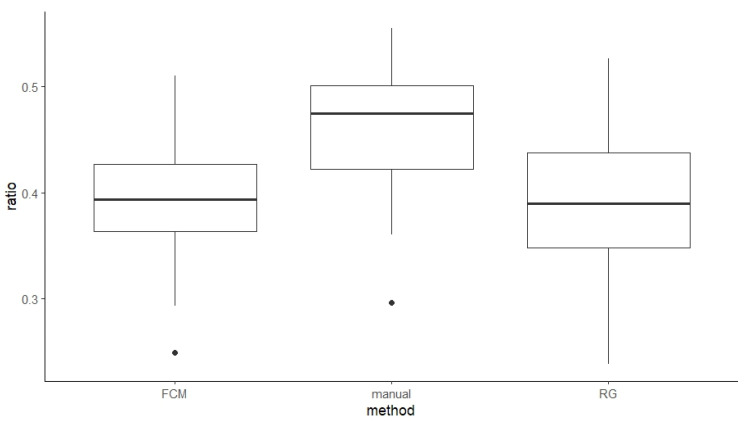
Comparison of NP-to-CSA ratios (%) derived from manual, FCM, and RG methods. (note, black dots indicate outliers within the data in boxplot charts).

Ratios derived from manual, FCM, and RG methods were 46%, 39%, and 38%, respectively (Figure 6). Tukey’s HSD tests revealed that ratios derived from manual measurements were significantly larger than the ones derived from FCM by 7.1% (*p* = 0.003) and RG by 7.7% (*p* = 0.001), respectively, while results derived from FCM and RG were not significantly different (*p* = 0.960).

**Figure 7:**
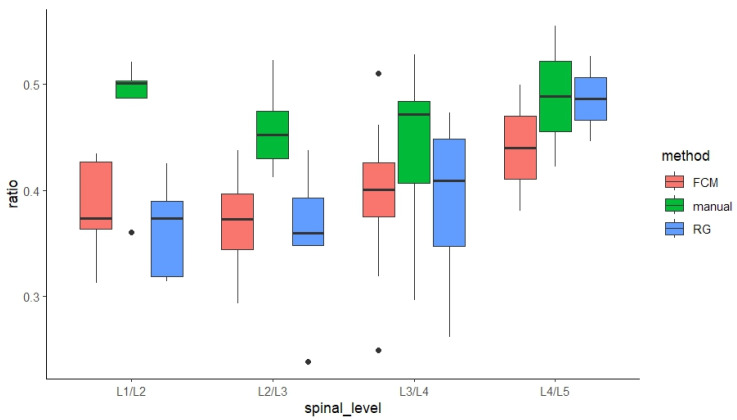
Comparison of NP-to-CSA ratios (%) derived from manual, FCM, and RG methods across four lumbar levels. (note, black dots indicate outliers within the data in boxplot charts).

In addition, similar results were found across all four spinal levels, where manual results were significantly larger than FCM- and RG-derived results (*p* < 0.001, Figure 7).

Based on the full dataset of MRI images (Table 3), the results derived from FCM and RG exhibited excellent agreement (PCC = 0.837, *p* < 0.001). On the other hand, pairwise comparison revealed a significant difference (i.e., 0.6% on average, *p* = 0.004). In addition, based on gender and spinal level, method-incurred differences were also evident. However, it should be noted that the mean absolute difference remained relatively small (≈0.6%). Results with significant differences were not necessarily associated with a more pronounced absolute difference. 

### 3.3. Influencing Factors of the NP-to-CSA Ratio

**Figure 8:**
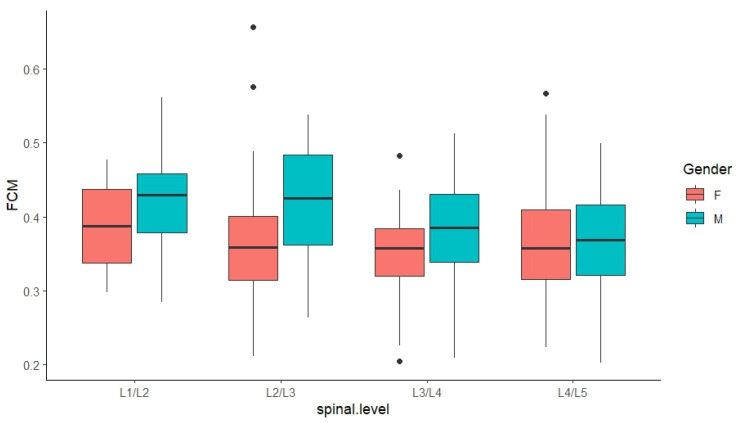
Comparison of FCM-derived NP-to-CSA ratios based on gender and spinal level. (note, black dots indicate outliers within the data in boxplot charts).

**Figure 9:**
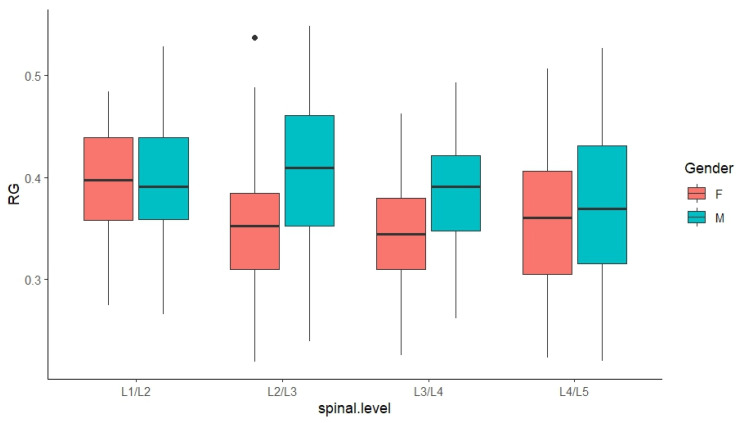
Comparison of RG-derived NP-to-CSA ratios based on gender and spinal level. (note, black dots indicate outliers within the data in boxplot charts).

Results from the ANOVA revealed a significant influence of gender (*p* < 0.001), spinal level (*p* < 0.05), and their interactions (*p* = 0.03) on the results derived from the FCM (Figure 8) and RG (Figure 9) methods, respectively. Since significant interaction terms were evident, additional analyses were performed and results were interpreted accordingly.

#### 3.3.1. FCM-Derived Results

As summarized in Table 4, based on FCM-derived measurements, at L2/L3 and L3/L4, NP-to-CSA ratios associated with male subjects were significantly larger by 6% (*p* < 0.001) and 3% (*p* < 0.05), respectively, while the difference at L1/L2 was also approaching significance (*p* = 0.080). When ratios were compared across multiple spinal levels, it was evident that male ratios differed significantly cranio-caudally along the lumbar region, with the largest ratio found at L1/L2 and L2/L3 at 42%, compared to 38% at the three lower levels. In contrast, female ratios remained relatively stable at around 36% across the lumbar region.

#### 3.3.2. RG-Derived Results

Based on RG-derived measurements (Table 5), a gender difference was evident and was more pronounced with male subjects by 6% at L2/L3 (*p* < 0.001), 3% at L3/L4 (*p* = 0.002), and 4% at L4/L5 (*p* = 0.012). However, the influence of spinal levels was more pronounced among females, where the ratio at L1/L2 (i.e., 40%) was significantly larger than the ones found for the rest of the lumbar region (i.e., 35%), except the one for L5/S1.

## 4. Discussion

The current study explored applications of MRI data to study morphological characteristics of the human lumbar IVDs. To the authors’ knowledge, this study may be the first attempt to quantitatively measure this internal morphological parameter, i.e., the NP-to-CSA ratio, which is a crucial input for FEM studies of spinal loadings and the associated biomechanical behaviors [1,2,4,5,30,31,32,33,34]. To establish the validity and practicality of the reported data, this study first included a relatively large sample of healthy IVDs after a thorough screening process, including the Pfirrmann grading system. Then, both manual (i.e., by a spine surgeon) and computer-assisted algorithms (i.e., FCM and RG) were applied to measure the morphology and composition of the internal structures. According to the original classification criteria, Grade I and II IVDs should have a clear distinction between the nucleus and anulus, while for Grade III IVDs, such a distinction becomes unclear [24]. This may pose challenges for manual contour tracing and measurement. In the current study, the results derived from the two manual measurements performed by the spine surgeon were, on average, off by 4% (i.e., 41% vs. 45%). In terms of absolute error, the resulting ratios were off by 10.5%. It was also noted that in extreme cases, the absolute error peaked at 56.5%. These findings suggest that manual measurement may lead to sizable over-/underestimation. Although previous studies using the manual method reported excellent reliability when measuring the peripheral characteristics of the spinal discs and paraspinal muscles (e.g., linear diameters and overall size) [7,11,21,22,23,35], it may be more difficult to maintain consistency when attempting to differentiate between the nucleus and anulus. This may partially explain the limited availability of data on the internal composition and morphology of spinal discs [8]. On the other hand, as the current study suggests, computer-assisted segmentation algorithms may be a reliable alternative. Since there is no “gold standard” method currently available to follow, two methods were proposed and assessed, i.e., FCM and RG, both of which have been widely used in other medical applications, such as tumor and blood vessel segmentation [15,16,17,19,20,36]. Based on the current sample, a mixture of healthy individuals and those who went to the hospital, computer-assisted algorithms were able to identify the nucleus while delivering excellent performance, which can be supported by the fact that 1) the results were within expectations as established in the literature (i.e., 30% to 50%) and 2) the actual difference between the two algorithms, although statistically significant, was indeed less relevant in terms of measurement accuracy (i.e., <1%). However, in terms of potential associations with other factors (i.e., gender and spinal level), subsequent analyses corresponding to each algorithm did exhibit slight differences in statistical interpretations. This may be attributed to the variability and uniqueness of the current sample, suggesting that further investigation should include a bigger and more diverse population and conduct more comprehensive statistical analyses and comparisons (e.g., applying a Bonferroni correction). In general, to address the scarcity of data and viable methodologies, it may still be helpful to establish the validity of using these two algorithms (or other computer-assisted methods in a similar capacity) to comprehensively quantify the internal morphology and composition of the spinal IVDs.

In the literature, morphological studies of spinal structures, including IVDs, vertebral endplates, vertebrae, paraspinal muscles, etc., have identified several influencing factors, including gender [7,21,37,38,39,40,41], age [40,41,42], and cephalocaudal changes (i.e., spinal levels) [7,11,43,44]. The current results are in support of the gender difference and cephalocaudal changes. It was evident that gender played a critical role in the internal morphology of the lumbar discs, particularly in the mid-lumbar region (i.e., L2/L3 to L3/L4), where male IVDs had a greater portion of the nucleus compared to the female ones. However, in the current sample, the two gender groups were also significantly different in body height and weight; therefore, the gender influence might have been confounded by these factors. In the literature, there has been evidence suggesting a correlation between gross anthropometry and spinal geometry [7,45,46]. IVDs in the upper and mid-lumbar regions had greater NP-to-CSA ratios compared to the ones in the lower lumbar region. However, this finding should be interpreted with caution regarding its generalizability as such trends/variability in NP-to-CSA ratios across the lumbar region may not be consistent. Unfortunately, besides the relatively simple methodology, the current study was also limited in further discussing the magnitude of the potential variability in NP-to-CSA ratios due to the disagreement between the results derived from the two computer-assisted algorithms. Based on the FCM-derived results, a cephalocaudally decreasing trend in the NP-to-CSA ratios was evident among male IVDs, while this ratio was stable among female IVDs. On the contrary, based on the RG-derived results, female IVDs were found to exhibit a cephalocaudal decrease in the ratio. Since pairwise comparisons suggested that the results derived from these two methods were very similar, one can speculate that the discrepancy in the corresponding cephalocaudal changes revealed may be attributed to the uniqueness of the data and the potential confounding factors. Therefore, future investigations should consider including more subjects with a diverse spectrum of demographic factors and applying a matched study design (e.g., matched for height, age, etc.) to improve the understanding of lumbar morphology and its associated factors.

The present study has several limitations, including the relatively small sample size. The screening criterion for age was designed to ensure the general health of the spinal discs and minimize the influence of disc degeneration. Unfortunately, this limited the current study’s ability to investigate the influence of age. In addition, no effort was made to achieve a gender- or body size-matched sample. The subjects presented a mixture of a convenience sample recruited from the university student body and historical medical records. Although this study did not screen MRI images based on scanning protocols/parameters, particularly section thickness, which varied from 3 to 4.5 mm, it did not investigate the potential influence of these MRI protocol parameters on the performance of computer-assisted methods, for example, how reliable the computer-assisted methods may be in handling MRI scans with a 3 mm section thickness vs. a 4.5 mm section thickness. Lastly, the current computer-assisted algorithms are among many capable tools for medical image segmentation and have therefore been pilot-tested for spinal morphology investigations. These limitations may help explain the discrepancy in the measurement results and the subsequent statistical results and interpretations.

## 5. Conclusions

The present study attempted to establish the validity of using computer-assisted methods to measure human lumbar morphology, particularly the internal composition (e.g., NP-to-CSA ratio), cephalocaudal changes, and gender influence, to address the limitations of previous studies and provide valuable morphological data regarding this ratio, which has been lacking in the literature. In comparison, the manual method may be susceptible to a sizable measurement error and may not be perceived as the sole data source for NP-to-CSA ratios. The influence of gender was significant. Significant craniocaudal changes, which appeared to interact with gender, were also noted. As mentioned above, this study suggests that comprehensive biomechanical models of the lumbar (e.g., FEMs) should consider the variability of spinal morphology and internal composition. Future studies should include larger sample sizes with greater variability in age and body size (e.g., height, weight, and BMI).

## Figures and Tables

**Table 1:** Demographic data for subjects included at each lumbar level.

			N	Mean	SD	Sig.
L1/L2	Age (years)	Female	21	29.7	5.2	0.730
		Male *	37	29.2	5.3	
	Ht (m)	Female	21	1.7	0.1	***<0.001***
		Male *	37	1.8	0.1	
	Wt (kg)	Female	21	74.0	14.9	***0.019***
		Male *	37	86.1	19.9	
	BMI (kg/m^2^)	Female	21	25.9	5.6	0.438
		Male *	37	27.1	5.3	
L2/L3	Age (years)	Female	55	28.7	5.4	0.801
		Male *	50	28.5	4.7	
	Ht (m)	Female	55	1.7	0.1	***<0.001***
		Male *	50	1.8	0.1	
	Wt (kg)	Female	55	69.5	18.4	***<0.001***
		Male *	50	82.7	19.0	
	BMI (kg/m^2^)	Female	55	25.2	5.8	0.490
		Male *	50	25.9	4.9	
L3/L4	Age (years)	Female	52	28.9	5.4	0.925
		Male *	50	29.0	4.9	
	Ht (m)	Female	52	1.7	0.1	***<0.001***
		Male *	50	1.8	0.1	
	Wt (kg)	Female	52	68.5	17.3	***<0.001***
		Male *	50	84.6	18.7	
	BMI (kg/m^2^)	Female	52	24.9	5.7	0.160
		Male *	50	26.4	5.0	
L4/L5	Age (years)	Female	39	28.6	5.4	0.492
		Male	34	29.4	4.4	
	Ht (m)	Female	39	1.7	0.1	***<0.001***
		Male	34	1.8	0.1	
	Wt (kg)	Female	39	72.5	18.3	***0.016***
		Male	34	83.4	19.4	
	BMI (kg/m^2^)	Female	39	26.0	5.9	0.682
		Male	34	26.6	5.4	
L5/S1	Age (years)	Female	36	28.5	5.3	0.654
		Male	23	29.1	5.1	
	Ht (m)	Female	36	1.7	0.1	***<0.001***
		Male	23	1.8	0.1	
	Wt (kg)	Female	36	71.7	19.2	***0.014***
		Male	23	82.8	10.7	
	BMI (kg/m^2^)	Female	36	25.5	6.2	0.561
		Male	23	26.3	3.0	

* One more male subject was included, despite the missing demographic data.

**Table 2:** NP-to-CSA ratios (%) derived from manual, FCM, and RG methods.

	Manual	FCM	RG
Ratio_NP-to-CSA_ (%)	46 ± 6	39 ± 6	38 ± 7

**Table 3:** Comparison of NP-to-CSA ratios (%) derived from FCM and RG methods based on gender and spinal level.

	**N**	**FCM**	**RG**	**Mean Absolute Difference**	***p* Value**
Gender					
Female	203	36 ± 7	36 ± 7	0.5	**0.078**
Male	197	40 ± 7	39 ± 7	0.7	***0.025***
Spinal level					
L1/L2	59	41 ± 6	40 ± 6	1.0	***0.037***
L2/L3	106	39 ± 8	38 ± 8	1.0	***0.031***
L3/L4	103	37 ± 6	36 ± 6	0.5	0.184
L4/L5	73	36 ± 7	36 ± 7	0.5	0.335
L5/S1	59	38 ± 7	37 ± 7	1.0	**0.076**
Total	400	38 ± 7	37 ± 7	0.6	***0.004***

**N**

**FCM**

**RG**

**Mean Absolute Difference**

***p* Value**

**Table 4:** Comparison of FCM-derived results across the lumbar region between two genders and across the lumbar region.

Spinal Level	Gender	N	FCM	L1/L2	L2/L3	L3/L4	L4/L5	L5/S1
L1/L2	F	21	**39** ± 5 *					
	M	38	**42** ± 7 *				***0.01***	
L2/L3	F	55	***36*** ± ***8 ^§^***					
	M	51	***42*** ± ***7 ^§^***			**0.072**	***0.005***	**0.091**
L3/L4	F	52	***35*** ± ***5***					
	M	51	***38*** ± ***6***					
L4/L5	F	39	35 ± 6					
	M	34	37 ± 7					
L5/S1	F	36	38 ± 8					
	M	23	38 ± 6					

* bold: indicates *p* < 0.10; ***^§^ italic and bold***: indicates *p* < 0.05.

**Table 5:** Comparison of RG-derived results across the lumbar region between two genders and across the lumbar region.

Spinal Level	Gender	N	RG	L1/L2	L2/L3	L3/L4	L4/L5	L5/S1
L1/L2	F	21	**40** ± 6 *		***0.046***	***0.021***	***0.025***	
	M	38	**40** ± 6 *					
L2/L3	F	55	***35*** ± ***7 ^§^***					
	M	51	***41*** ± ***7 ^§^***					***0.036***
L3/L4	F	52	***35*** ± ***5***					
	M	51	***38*** ± ***5***					
L4/L5	F	39	***34*** ± ***6***					
	M	34	***38*** ± ***7***					
L5/S1	F	36	37 ± 7					
	M	23	36 ± 7					

* bold: indicates *p* < 0.10; ***^§^ italic and bold***: indicates *p* < 0.05.

## Data Availability

The data are available from the corresponding author(s) upon reasonable request.
